# Effect of aerobic and resistance exercise on the mitochondrial peptide MOTS-c in Hispanic and Non-Hispanic White breast cancer survivors

**DOI:** 10.1038/s41598-021-96419-z

**Published:** 2021-08-19

**Authors:** Christina M. Dieli-Conwright, Nathalie Sami, Mary K. Norris, Junxiang Wan, Hiroshi Kumagai, Su-Jeong Kim, Pinchas Cohen

**Affiliations:** 1 Division of Population Sciences, Department of Medical Oncology, Dana-Farber Cancer Institute, Harvard Medical School, 375 Longwood Avenue, Boston, MA 02215 USA; 2 Department of Medicine, Keck School of Medicine, University of Southern California (USC), Los Angeles, CA 90033 USA; 3 Leonard Davis School of Gerontology, USC, Los Angeles, CA 90033 USA

**Keywords:** Biomarkers, Oncology

## Abstract

MOTS-c is a mitochondrial derived peptide with exercise mimetic activity that elicits beneficial effects on metabolism and exercise capacity. Furthermore, MOTS-c effects in humans are affected by race, potentially via ethnic-specific mtDNA variations. Women treated for breast cancer are at an increased risk for cardiovascular disease, diabetes and obesity, due to side effects of cancer-treatments. We conducted a secondary analysis of the effects of a 16-week aerobic and resistance exercise intervention on MOTS-c in Hispanic and Non-Hispanic White breast cancer survivors (BCS). BCS (Stage I–III) were randomized to exercise or standard care. The intervention promoted aerobic and resistance exercise for 16 weeks. MOTS-c was analyzed in fasting plasma using an in-house ELISA. Within and between group differences were assessed by paired t-test and repeated measures ANOVA. Pearson’s correlation was computed to assess the association between MOTS-c and metabolic biomarkers at baseline and post-exercise. Twenty-five Hispanic-BCS and 24 non-Hispanic White BCS were included. Hispanic BCS were younger, of greater adiposity, had higher stage cancers, and had worse metabolic profiles at baseline compared to non-Hispanic White BCS (p < 0.001). Post-exercise, MOTS-c levels significantly increased when compared to baseline and the usual care group among non-Hispanic White BCS (p < 0.01) but not among Hispanic breast cancer survivors (p > 0.01). Post-exercise levels of MOTS-c among non-Hispanic White BCS were significantly associated with reductions in fat mass, body weight, HOMA-IR, CRP, and an increase in lean mass (p < 0.01). A 16-week aerobic and resistance intervention increased MOTS-c levels among non-Hispanic White BCS.

**Trial registration:** This trial is registered on ClinicalTrials.gov: NCT01140282 as of June 9, 2010. https://clinicaltrials.gov/ct2/show/NCT01140282.

## Introduction

Mitochondrial-derived peptides (MDPs) are peptides encoded by mitochondrial DNA, have similar energetic functions to mitochondria, and play a cytoprotective role in the body^1^. MOTS-c is a MDP with exercise mimetic activity that elicits beneficial effects on metabolism and exercise capacity^2^. MOTS-c stimulates glucose utilization, fat-oxidization, reduces inflammation, and protects against experimental models of metabolic disease^3^. Furthermore, MOTS-c effects in humans are affected by race, potentially via ethnic-specific mtDNA variations^4^. Women treated for breast cancer are at an increased risk for cardiovascular disease (CVD), diabetes and obesity, due to side effects of cancer-treatments^5^. Exercise mitigates many of these side effects^6^, but the role of MOTS-c regulation by exercise among breast cancer survivors (BCS) of varying ethnicities is unknown. Hispanics, in particular, suffer from more comorbid conditions including obesity, diabetes, and heart disease^7^. Given that increased research on MDPs has shown their involvement in pathological changes of CVD through different mechanisms^1^, the impact of exercise on MOTS-c is of specific interest in this particular at-risk demographic encompassing cancer survivors and minority women.

The overall purpose of this trial was to compare a 16-week supervised moderate-vigorous intensity aerobic and resistance exercise intervention to usual care in physically inactive, Hispanic and non-Hispanic White BCS. We previously reported that the exercise intervention led to significant improvements in metabolic syndrome, sarcopenic obesity, and circulating biomarkers, muscle strength, psychosocial health that were maintained at 3-month follow-up^8^. Here, we report the secondary outcome of MOTS-c. We hypothesized that a combined exercise intervention performed within 6 months of cancer treatment completion would increase MOTS-c in both Hispanic and non-Hispanic White BCS, and explored the presence of ethnic differences in MOTS-c.

## Methods

### Participants/consent

Eligible participants were < 6 months post-treatment for chemo- or radiation-therapy for stage 0–III breast cancer and were non-smokers, physically inactive (< 60 min of structured exercise/week), with BMI ≥ 25.0 kg/m^2^ (or body fat > 30%) and waist circumference > 88 cm. Participants were verbally screened for eligibility by phone or in person at time of consent. Treatment history and diagnosis were confirmed by medical record abstraction. Body composition measure were obtained at time of screening per testing methods described below (Covariate Measures).

Recruitment occurred between August 1, 2012 and December 31, 2016 from the USC Norris Comprehensive Cancer Center and Los Angeles County Hospital. The protocol and informed consent were IRB-approved (HS-12-00141) and registered (ClinicalTrials.gov: NCT01140282). A signed informed consent was obtained from each participant. Participants were randomized to exercise or usual care following the completion of baseline testing using concealed randomization lists.

### Experimental design

Detailed methods^9^, and primary outcomes related to metabolic syndrome were published previously^10^. Endpoints were assessed at baseline, post-intervention (month 4), and 3-month follow-up (exercise group only). To enhance participation, usual care participants were offered the exercise program following the study period. This secondary analysis examined a progressive combined (aerobic and resistance) exercise intervention on MOTS-c in Hispanic versus Non-Hispanic White BCS.

### Blood collection and analysis

Fasting (~ 12 h) blood (~ 30 cc) was obtained from the antecubital vein by trained phlebotomists. Plasma was stored at − 80° Celsius until batch analysis at study completion.

### Mitochondrial open-reading-frame of the twelve S rRNA-type c (MOTS-c)

Mitochondrial-derived peptide MOTS-c was assessed through fasting blood draws obtained at baseline and post-intervention (4 months) and analyzed in plasma using an in-house ELISA^3^. Briefly, MOTS-c was extracted in 90% acetonitrile and 10% 1 N HCl prior to assay. A 96-well microtiter plate was coated with rabbit anti-MOTS-c polyclonal antibody (Yenzym Antibodies, LLC, South San Francisco, CA) at 0.5 μg/well and incubated on a shaker. Standards, controls or extracted samples and pre-titered detection antibody were added to the appropriate wells and incubated overnight after 2 washes with wash buffer and 2 washes with Superblock buffer (Pierce Chemicals, Rockford, IL). Wells were then added streptavidin-HRP conjugate after wash and further incubated for 30 min at room temperature. After 4 washes with wash buffer, 200 μl/well of 1-step ultra TMB will be added and incubated for 10–20 min. The reaction was terminated by the addition of 2 N H_2_SO_4_, and absorbance was measured on a plate spectrophotometer (Molecular Designs, Sunnyvale, CA) at 490 nm. The intra- and inter-assay coefficient variations were less than 10%.

### Cardiometabolic analysis

Glucose and glycosylated hemoglobin (HbA1c) were measured on a Vitros 4600 Analyzer (Ortho Clinical Diagnostics, Rochester, NY). High-sensitivity C-reactive protein was measured by immunoturbidmetric assay. Enzyme-linked immunosorbent assays were used to measure insulin, IL-6, IL-8, TNF-a, leptin, and adiponectin. Homeostasis model assessment (HOMA-IR) was used to estimate insulin resistance using the validated equation: Fasting Plasma Insulin × Fasting Plasma Glucose (mmol/l)/22.5 (needs ref). Duplicate testing was performed with coefficients of variation for all samples < 10%.

### Cardiorespiratory fitness

A single-stage submaximal treadmill test was used to estimate maximal oxygen uptake, VO_2max_^11^. Participants first performed a 4-min warm up by walking on a treadmill (Desmo Woodway, Waukesha, WI) at a speed (2.0, 3.0, 4.0, or 4.5 mph) that increased their heart rate between 50 and 70% heart rate maximum. This was followed by the 4-min test at the same speed with a 5% grade; heart rate was measured during the final 30 s of the test. Using heart rate, speed, age and gender, estimated maximal oxygen uptake was predicted using the test-specific regression formula^11^.

### Muscular strength

Estimated maximal voluntary strength (1-RM) was assessed for the chest press, latissimus pulldown, knee extension, and knee flexion using the 10-repetition maximum (10-RM) method (Tuff Stuff, Pomona, CA)^12^. Participants completed a warm-up load of ~ 5–8-RM before attempting 10-RM. A 2-min rest period was given between attempts; 3–5 attempts were performed.

### Dual energy X-ray absorptiometry (DXA)

Whole body DXA scans were used to assess fat-free mass, fat mass, and body fat % (Lunar GE iDXA, Fairfield, Connecticut).

### Covariate measures

Weight was measured to the nearest 0.1 kg on an electronic scale with the patient wearing a hospital gown and no shoes and height was measured to the nearest 0.5 cm with a fixed stadiometer in order to calculate BMI. Waist circumference was measured at the midpoint between the lower margin of the last palpable rib and the iliac crest. Physical activity history was assessed at baseline using an interviewer-administered, validated questionnaire to assess historical, past-year, and past-week physical activity^13^. Three-day dietary records (2 weekdays and 1 weekend day) were completed at baseline, post-intervention, and 3-month follow-up (exercise group only) within 1-week of each assessment and analyzed using Nutritionist Pro™ (Woodinville, WA). Participants completed the Charlson Comorbidity questionnaire^14^. Cancer-related information (i.e., time since treatment completion, time since diagnosis, disease stage, hormone-receptor status, endocrine therapy, and surgery) was abstracted from medical records.

### Exercise intervention

The exercise program aligned with American Cancer Society/American College of Sports Medicine (ACS/ACSM) exercise guidelines for cancer survivors (150 min of aerobic exercise and 2–3 days of resistance exercise training/week)^15^. Participants received three supervised one-on-one exercise sessions/week. Days one and three consisted of aerobic and resistance exercise of ~ 80 min and Day two included ~ 50 min of aerobic exercise. All sessions were led by a certified ACS/ACSM Cancer Exercise Trainer. Participants wore a Polar® heart monitor (Lake Success, NY) during each exercise session. Each session began with a 5-min aerobic exercise warm-up at 40–50% estimated VO_2max_. Sequenced resistance exercise followed in circuit training fashion with no rest periods between exercises: Leg Press ⇔ Chest Press ⇒ Lunges ⇔ Seated Row ⇒ Leg Extensions ⇔ Triceps Extensions ⇒ Leg Flexion ⇔ Biceps Curl; where ⇔ indicates the two exercises that alternated until all sets were completed, then the following pair of exercises was performed. Initial resistance was set at 80% of the estimated 1-RM for lower body exercises and 60% estimated 1-RM for upper body exercises. When the participant was able to complete three sets of 10 repetitions at the set weight in two consecutive sessions then the weight is increased by 10%. Repetitions increased from 10 (week 4) to 12 (week 8) to 15 (week 12) every 4 weeks to safely build muscular endurance. Compression garments were required during the exercise sessions for all participants who held prescriptions.

Resistance exercises were followed by self-selected aerobic exercise: treadmill walking/running; rowing machine; stationary bicycle. Heart rate (HR) was monitored throughout the aerobic sessions to maintain a HR at 65–80% of maximum HR. Target HR was increased every four weeks to safely build cardiorespiratory endurance and to maintain the prescribed intensity as participants improved their cardiorespiratory fitness. Duration of the aerobic sessions was increased from 30 min (week 1) to 50 min (week 16) as cardiorespiratory fitness increased to meet the exercise guidelines for cancer survivors. Participants ended each session with a 5-min cool down at 40–50% estimated VO_2max_. The trainers documented attendance and minutes of exercise per session.

### Statistical analyses

As this is a secondary analysis of the parent trial which focused on metabolic syndrome, the sample size was based on projected changes in insulin^16^. Enrollment of 100 women provided 80% statistical power (α = 0.05) to detect a 2.6 μU/ml (SD = 4.0 μU/ml) difference in mean insulin levels assuming 20% drop-out using a two-group t-test.

Within-group differences in mean changes for individual outcomes measured at post-intervention were evaluated using general linear models repeated-measures ANOVAs. Between-group differences in mean changes for individual outcomes measured at post-intervention were evaluated using mixed-model repeated measure analysis. A priori covariates included type of treatment (chemotherapy, radiation, or both), surgery type, time on hormonal therapy, comorbidities, age and BMI were explored in models due to their possible associations with outcomes, but none modified results. The association between MOTS-c and physical fitness, muscle strength, and cardiometabolic biomarkers was computed at baseline for both groups and changes in these variable post-exercise in the exercise group using Pearson correlation.

Post-hoc analyses included stratification by menopausal status at time of diagnosis (women were classified as postmenopausal if amenorrheic over the previous 12 months). Analyses were performed using SAS (Version 9.4, Cary, NC).

### Ethics approval and consent to participate

The protocol and informed consent were approved (HS-12-00141) by the USC Institutional Review Board.

### Consent for publication

A signed informed consent was obtained from each participant. The informed consent is available upon request.

## Results

The study CONSORT diagram is reported elsewhere^10^. Briefly, we assessed 418 women for eligibility of which 100 were randomized to the exercise or usual care group. Four participants in the exercise group and five participants in the usual care group did not complete the study. Baseline characteristics also are reported elsewhere^10^ and were similar across the two groups. For this analysis, 25 Hispanic BCS and 24 non-Hispanic White BCS with BMI of 33.5 ± 5.5 kg/m^2^ and whom had adequate blood samples available were included. Hispanic BCS were younger, of greater adiposity, had higher stage cancers, and had worse metabolic profiles at baseline compared to non-Hispanic White BCS (p < 0.001).

### Baseline MOTS-c correlations

Baseline levels of MOTS-c among Hispanic and non-Hispanic White BCS were significantly associated with anthropometric measures including weight, fat mass, waist circumference, fitness (VO_2max_ and muscular strength), and cardiometabolic biomarkers (p < 0.01; Table 1). Significant correlations were observed for biomarkers of glucose metabolism and inflammation among non-Hispanic White BCS, but not among Hispanic BCS (p > 0.05).

**Table 1:** Baseline correlations of MOTS-c and anthropometrics, fitness, and cardiometabolic biomarkers among Hispanic and Non-Hispanic BCS.

	Hispanic	Non-Hispanic
**MOTS-c**		
Weight, kg	− 0.60 (0.001)	− 0.69 (0.002)
Lean mass, kg	0.59 (0.003)	0.77 (< 0.001)
Fat mass, kg	− 0.81 (< 0.001)	− 0.86 (< 0.001)
Body fat, %	− 0.77 (< 0.001)	− 0.73 (< 0.001)
Waist circumference, cm	0.69 (0.001)	− 0.80 (< 0.001)
Hip circumference, cm	0.70 (0.001)	− 0.79 (< 0.001)
VO_2max_, kg/ml/min	0.90 (< 0.001)	0.91 (< 0.001)
Leg extension, kg	0.89 (< 0.001)	0.93 (< 0.001)
Leg flexion, kg	0.85 (< 0.001)	0.86 (< 0.001)
Chest press, kg	0.75 (< 0.001)	0.77 (< 0.001)
Latissimus pull, kg	0.76 (< 0.001)	0.78 (< 0.001)
Glucose, mg/dl	− 0.45 (> 0.05)	− 0.78 (< 0.001)
Insulin, uIU/ml	− 0.33 (> 0.05)	− 0.80 (< 0.001)
HOMA-IR	− 0.46 (> 0.05)	− 0.83 (< 0.001)
Total cholesterol, mg/dl	− 0.72 (< 0.001)	− 0.75 (< 0.001)
LDL, mg/dl	− 0.67 (0.001)	− 0.69 (0.001)
HDL, mg/dl	− 0.73 (< 0.001)	0.83 (< 0.001)
Triglycerides, mg/dl	− 0.80 (< 0.001)	− 0.84 (< 0.001)
HgA1c (%)	− 0.28 (> 0.05)	− 0.90 (< 0.001)
CRP, mg/dl	− 0.40 (> 0.05)	− 0.75 (< 0.001)
Leptin, ng/ml	− 0.21 (> 0.05)	− 0.41 (0.10)

### MOTS-c

**Figure 1:**
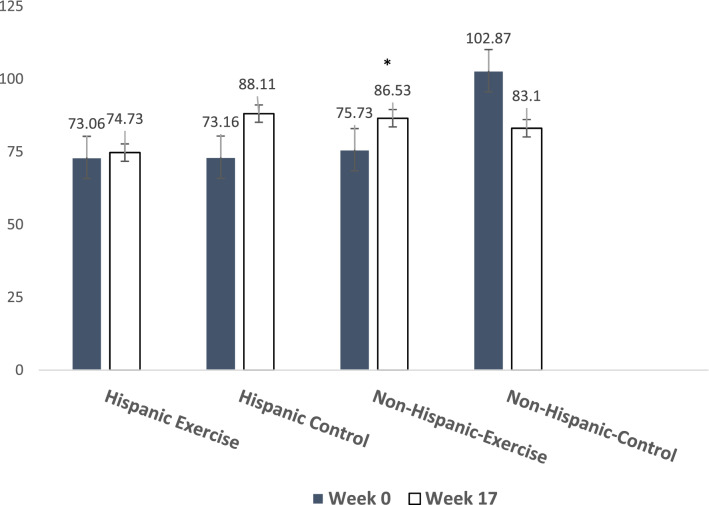
Exercise-induced changes in MOTS-c among Hispanic and Non-Hispanic breast cancer survivors. *Significance difference from baseline, p < 0.01.

MOTS-c outcomes are displayed in Fig. 1. Post-exercise, MOTS-c levels significantly increased when compared to baseline in the usual care group among non-Hispanic White BCS (p < 0.01). MOTS-c did not significantly change following exercise among Hispanic BCS (p > 0.05).

### Post-exercise MOTS-c correlations

Post-exercise changes in MOTS-c among non-Hispanic White BCS were significantly associated with multiple anthropometric measures including reductions in fat mass, waist circumference, BMI, body weight, HOMA-IR, CRP, and an increase in lean mass (p < 0.01; Table 2); fitness measures including increased muscle strength and VO_2max_ (p < 0.01); and cardiometabolic biomarkers such as reductions in glucose, insulin, HOMA-IR, total cholesterol, LDL, triglycerides, HgA1c, CRP, leptin, and increased HDL (p < 0.01). Significant correlations were observed for fitness measures among Hispanic BCS (< 0.001).

**Table 2:** Post-intervention correlations of changes in MOTS-c and anthropometrics, fitness, and cardiometabolic biomarkers among Hispanic and Non-Hispanic BCS.

	Hispanic	Non-Hispanic
**MOTS-c**		
Weight, kg	− 0.45 (> 0.05)	− 0.71 (0.002)
Lean mass, kg	0.31 (> 0.05)	0.65 (0.002)
Fat mass, kg	− 0.45 (> 0.05)	− 0.83 (< 0.001)
Body fat, %	− 0.37 (> 0.05)	− 0.87 (< 0.001)
Waist circumference, cm	− 0.58 (0.004)	− 0.81 (< 0.001)
Hip circumference, cm	− 0.65 (0.004)	− 0.80 (< 0.001)
VO_2max_, kg/ml/min	0.87 (< 0.001)	0.95 (< 0.001)
Leg extension, kg	0.81 (< 0.001)	0.90 (< 0.001)
Leg flexion, kg	0.83 (< 0.001)	0.85 (< 0.001)
Chest press, kgs	0.76 (< 0.001)	0.79 (< 0.001)
Latissimus pull, kg	0.73 (< 0.001)	0.79 (< 0.001)
Glucose, mg/dl	− 0.35 (> 0.05)	− 0.77 (< 0.001)
Insulin, uIU/ml	− 0.29 (> 0.05)	− 0.79 (< 0.001)
HOMA-IR	− 0.34 (> 0.05)	− 0.85 (< 0.001)
Total cholesterol, mg/dl	− 0.42 (> 0.05)	− 0.78 (< 0.001)
LDL, mg/dl	− 0.39 (> 0.05)	− 0.79 (< 0.001)
HDL, mg/dl	0.24 (> 0.05)	0.80 (< 0.001)
Triglycerides, mg/dl	− 0.16 (> 0.05)	− 0.84 (< 0.001)
HgA1c (%)	− 0.18 (> 0.05)	− 0.89 (< 0.001)
CRP, mg/dl	− 0.30 (> 0.05)	− 0.76 (< 0.001)
Leptin, ng/ml	− 0.19 (> 0.05)	− 0.45 (0.10)

## Discussion

A supervised 16-week aerobic and resistance exercise intervention designed to improve metabolic syndrome also led to significant improvements in MOTS-c in non-Hispanic White BCS. This is the first study to our knowledge to report improvements in MOTS-c with a structured combined exercise intervention in an ethnically-diverse sample of overweight or obese BCS soon after treatment.

Here we confirm an exercise-induced increase in MOTS-c among non-Hispanic White BCS, as noted in adult populations. Few studies have been developed in the area of exercise and MOTS-c^17^, yet there is growing interest in the effects of exercise on MDPs in human subjects. Reynolds et al. (2019) reported significant increases in MOTS-c in response to exercise^18^. While these results are similar to what we reported in the non-Hispanic White BCS group, associations are hard to draw as Reynolds et al. (2019) involved a small sample size (n = 10) of healthy young males^18^. The intervention was also of a much shorter duration, involving only one bout of high intensity cycling. Participants completed ten, 60-s intervals at peak power workloads (determined from visit one), followed by 75-s of rest/low intensity cycling. Blood and muscle samples were taken before, immediately after, and 4-h after recovery^18^. Overall, there is a severe lack of conclusive evidence regarding the effects of exercise on MOTS-c among adults with a history of cancer. However, additional work has been done with another MDPs, Humanin. The largest exercise trial, to our knowledge, involving Humanin was done in prediabetic men (n = 55) with impaired glucose regulation (IGR). Gidlund et al. (2016) reported for the first time, that Humanin protein levels increase in human skeletal muscle following a 12-week resistance training intervention. This was a three-armed trial in that participants were either randomized to resistance training, Nordic walking, or a control group. Participants exercised three times per week (60 min/session), with the exercise intensity and load progressively increasing every 4 weeks. Blood and tissue samples were taken before and after the intervention. Gidlund et al. (2016) reported that skeletal muscle humanin protein levels increased after resistance training, however no change in humanin protein levels was seen in serum in any of the intervention groups^19^.

This evidence is compelling in regard to the breast cancer population as there has been associations between MDPs and cardiovascular risk factors, such as aging, hyperlipidemia, insulin resistance, and atherosclerosis^1^. MDPs play a cardioprotective role by intervening in these risk factors through reducing oxidative stress, influencing metabolism of glycolipids, increasing glucose utilization and insulin sensitivity, and protecting endothelial function^1^. BCS are at elevated risk of multiple comorbid conditions. Part of this is natural as risk of comorbid conditions increase with age and most breast cancer patients are commonly diagnosed after 50 years of age^5^. However, this group is also most susceptible to cardiovascular disease (CVD) and developing metabolic syndrome (MetS)^5^. This could be due to a multitude of effects, such as inadequate diet, sedentary behavior, and consequential weight gain, but also cancer treatments with secondary cardiotoxic and atheromatous effects^5^. Some breast cancer treatments, such as chemotherapies with anthracyclines and trastuzumab or radiation, can increase risk of cardiac dysfunction^5^.

In mouse models, Humanin seems to have a role in the prevention of age-related diseases such as type 2 diabetes and potentially cardiovascular diseases. However, any potential exercise-mediated protective and risk reducing effects need to be further validated^19^. Yet, this evidence is still promising that there is a connection and potential for exercise to influence MDPs and thus, impact comorbid conditions, not only in mouse and human subjects, but specifically cancer survivors. While there is speculation that MOTS-c is involved in exercise adaptation, there might be similarities between Humanin and MOTS-c to indicate that they act in concert since their signaling pathways are similar^19^. Given the fact that MOTS-c has been shown to target skeletal muscle and enhance glucose metabolism, there is implications that MOTS-c could assist in regulating obesity, diabetes, exercise, and longevity^2^, making it a novel marker to study in the topic of exercise oncology.

In alignment with the positive impact of exercise on MOTS-c among non-Hispanic White BCS, MOTS-c was positively correlated with increases in lean mass and fat loss, implying that as lean mass increases and fat mass decreases, MOTS-c increases. These positive results support previous MOTS-c observational studies demonstrating associations with MOTS-c and biomarkers of skeletal muscle both in plasms and tissue samples^2,20^. Additionally, post-exercise MOTS-c levels were associated with improved cardiometabolic biomarkers contributing to the overall positive impact of exercise on a population of cancer survivors highly prone to cardiovascular-related co-morbidities.

Notably, our results highlight the identification of a possible ethnicity-dependent relationship whereby Hispanic BCS did not experience improvements in MOTS-c following the exercise intervention. However, at baseline, measures of physical fitness were positively associated with MOTS-c, as expected, for both Hispanic and non-Hispanic White BCS. As this is the first trial to examine MOTS-c in cancer survivors, it is difficult to ascertain potential mediators of ethnic differences in response to exercise, which may include socio-cultural influences, therefore future works elicits a focus on observational data surrounding ethnic profiles of MOTS-c among cancer survivors, and subsequent exercise interventions to improve MOTS-c. The question remains as to whether there is an underlying impairment of mitochondrial biogenesis associated with obesity and metabolic profiles, of which are worsened when compared to non-Hispanic Whites, among Hispanic BCS that may alter the responsiveness of MOTS-c to exercise.

Strengths of our study include the focus on high-risk BCS with high rates of inactivity and obesity, targeting the early survivorship period, the ethnically-diverse sample, the randomized controlled trial design, the high adherence rate, and the modest loss-to-follow up. Limitations include small sample size, lack of direct physical function and physical fitness measures (i.e., 1-RM and VO_2max_), and lack of an attention control group.

## Conclusions

In summary, a 16-week aerobic and resistance intervention increased MOTS-c levels among non-Hispanic White BCS but not Hispanic BCS. Non-Hispanic White BCS who experience exercise-induced improvements in MOTS-c may also experience improved insulin sensitivity and body composition thereby reducing the risk for comorbid conditions. Despite poorer metabolic profiles, Hispanic BCS may not experience exercise-induced benefits on MOTS-c. Future trials should aim to explore potential ethnic differences in MOTS-c levels following exercise among cancer survivors.

## Data Availability

The datasets used and/or analyzed during the current study are available from the corresponding author on reasonable request.
